# Extremely Low Frequency Magnetic Fields Do Not Affect LTP-Like Plasticity in Healthy Humans

**DOI:** 10.3389/fnhum.2020.00014

**Published:** 2020-02-05

**Authors:** Fioravante Capone, Giovanni Pellegrino, Francesco Motolese, Mariagrazia Rossi, Gabriella Musumeci, Vincenzo Di Lazzaro

**Affiliations:** ^1^Unit of Neurology, Neurophysiology, and Neurobiology, Department of Medicine, Università Campus Bio-Medico di Roma, Rome, Italy; ^2^NeXT: Neurophysiology and Neuroengineering of Human-Technology Interaction Research Unit, Campus Bio-Medico University, Rome, Italy; ^3^Neurology and Neurosurgery, Montreal Neurological Institute and Hospital, McGill University, Montreal, QC, Canada

**Keywords:** magnetic fields, brain stimulation, plasticity, long-term potentiation, low frequency, extremely low-frequency magnetic fields

## Abstract

**Introduction:**

Several studies explored the biological effects of extremely low-frequency magnetic fields (ELF-MFs) *in vitro*, reporting the induction of functional changes in neuronal activity. In particular, ELF-MFs can influence synaptic plasticity both *in vitro* and in animal models but some studies reported an increase in long-term potentiation (LTP) whereas others suggested its reduction. However, no specific study has investigated such effect on humans.

**Aims:**

To evaluate whether ELF-MFs affect the propensity of the human cortex to undergo LTP-like plasticity.

**Methods:**

We designed a randomized, single-blind, sham-controlled, cross-over study on 10 healthy subjects. Cortical plasticity was induced by intermittent theta burst stimulation (iTBS) before and after 45-min ELF-MFs (75 Hz; 1.8 mT) or sham exposure and was estimated by measuring the changes of motor evoked potentials (MEP) amplitude before and after each iTBS.

**Results:**

No adverse events were reported. No significant effects of ELF-MFs on cortical plasticity were found.

**Conclusion:**

Whole-brain exposure to ELF-MFs (75 Hz; 1.8 mT) is safe and does not seem to significantly affect LTP-like plasticity in human motor cortex.

## Introduction

Several studies explored the biological effects of extremely low-frequency (0–300 Hz) magnetic fields (ELF-MFs) *in vitro*, reporting the induction of functional changes in neuronal activity (Di Lazzaro et al., 2013). On human subjects, ELF-MFs can produce measurable changes in brain electrical activity and can also influence cerebral functions such as motor control, sensory perception, cognitive activities, sleep, and mood (Bagheri Hosseinabadi et al., 2019). More recently, the potential application of ELF-MFs for non-invasively modulating brain activity has been investigated in different neuropsychiatric diseases such as stroke (Capone et al., 2009) and depression (Bagheri Hosseinabadi et al., 2019).

According to the most part of the studies, ELF-MFs are safe and well tolerated (Di Lazzaro et al., 2013). However, previous papers have showed deterioration in memory and learning processes after ELF-MFs exposure both in animals (Jadidi et al., 2007) and in humans (Podd et al., 2002; Corbacio et al., 2011). Instead, other studies have reported, after chronic ELF-MFs exposure, a positive effect in social recognition memory (Varró et al., 2009) and spatial learning (Liu et al., 2015).

Long-term potentiation (LTP) is a form of synaptic plasticity (Bear and Malenka, 1994) and is considered one of the most important molecular mechanisms underlying learning and memory. ELF-MFs affect synaptic plasticity both *in vitro* (Ahmed and Wieraszko, 2008; Varró et al., 2009; Balassa et al., 2013) and in animal models (Komaki et al., 2014), but type and significance of such effect remain unclear. Indeed, some studies reported an increase of LTP (Ahmed and Wieraszko, 2008; Komaki et al., 2014) whereas others suggested its reduction (Balassa et al., 2013). Moreover, no specific study has investigated such effect in humans.

Recently, protocols of repetitive transcranial magnetic stimulation (rTMS) of the brain resembling experimental LTP models have been introduced. The rTMS paradigm known as intermittent theta burst stimulation (iTBS) produces a prolonged increase of cortical excitability (Huang et al., 2005). The effects of iTBS are influenced by drugs that act at the N-methyl-D-aspartic acid (NMDA) receptor level (Huang et al., 2007), supporting the hypothesis that the after-effects of iTBS involve LTP-like changes. Thus, by this technique, it is possible to evaluate, non-invasively, synaptic plasticity in humans.

Aim of the present study was to evaluate, by means of iTBS, whether ELF-MFs exposure affects the propensity of the cortex to undergo LTP-like plasticity.

## Materials and Methods

We designed a randomized, single-blind, sham-controlled, cross-over study on 10 healthy subjects (7 F, 25 ± 2 years). The study was performed according to the Declaration of Helsinki, was approved by the Local Ethics Committee and all participants signed a written informed consent.

### Study Design

**FIGURE 1:**
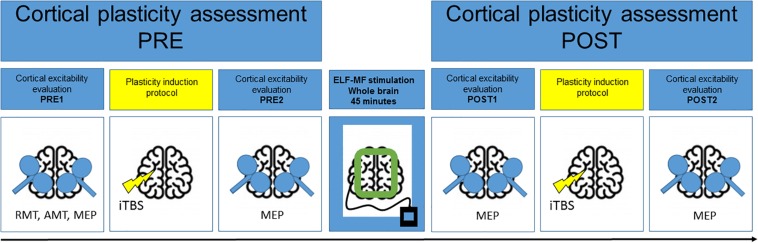
Experimental design. Cortical plasticity was induced by iTBS before and after 45-min ELF-MFs/sham exposure and was estimated by measuring the changes of motor evoked potentials (MEP) amplitude before and after each iTBS.

Figure 1 graphically depicts the experimental design. Cortical plasticity was estimated by measuring the changes of cortical excitability induced by iTBS before and after 45 min of exposure to ELF-MFs or to sham stimulation. All the subjects underwent two sessions of the study (REAL or SHAM) at a distance of at least 1 week. The order of the session was counterbalanced between subjects.

### Evaluation of Cortical Plasticity

For the evaluation of cortical excitability, magnetic stimulation was performed with a high-power Magstim 200 (Magstim Co., Whitland, Dyfed, United Kingdom). A figure-of-eight coil, with external loop diameter of 9 cm, was held over the motor cortex at the optimum scalp position to elicit motor evoked potentials (MEPs) in the contralateral first dorsal interosseous muscle (FDI). The induced current flowed in a postero-anterior direction. MEPs were recorded via two 9-mm-diameter Ag–AgCl surface electrodes with the active electrode over the motor point of the FDI and the reference on the metacarpophalangeal joint of the index finger. The EMG was amplified and filtered (bandwidth 3 Hz–3 kHz) by D360 amplifiers (Digitimer, Welwyn Garden City, Herts, United Kingdom). Data were collected on a computer with a sampling rate of 10 kHz per channel and stored for later analysis using a CED 1401 A/D converter (Cambridge Electronic Design, Cambridge, United Kingdom).

At baseline, we measured motor thresholds and MEP amplitude bilaterally. Resting motor threshold (RMT) was defined as the minimum stimulus intensity that produced a liminal MEP (about 50 μV in 50% of ten trials) at rest. Active motor threshold (AMT) was defined as the minimum stimulus intensity that produced a liminal MEP (about 200 μV in 50% of ten trials) during isometric contraction of the tested muscle. A constant level of voluntary contraction was maintained with reference to an oscilloscope display of the EMG signal in front of the subject. Auditory feedback of the EMG activity was also provided. RMT and AMT are given in percentage of maximum stimulator output (% MSO). For MEP amplitude, the responses to 15 stimuli at an intensity of 120% RMT were averaged at rest (Di Lazzaro et al., 2008, 2011). Trials contaminated by EMG activity were discarded.

Intermittent theta burst stimulation was delivered over the right motor cortex “hotspot” for MEPs in the contralateral FDI muscle using a DUOMAG XT stimulator (DEYMED Diagnostic, Czech Republic) and a figure-of-eight shaped coil, with the handle pointed posteriorly and approximately perpendicular to the central sulcus. The initial direction of the current induced in the brain was anterior to posterior. The magnetic stimulus had a biphasic waveform with a pulse width of about 280 μs and maximum magnetic field strength of 1.5 T. The stimulation intensity was defined in relation to AMT evaluated using the MagPro stimulator. An intensity of 80% AMT was used. We used the iTBS protocol in which 10 bursts of high frequency stimulation (3 pulses at 50 Hz) are applied at 5 Hz every 10 s for a total of 600 pulses (Huang et al., 2005).

### ELF-MFs Exposure

The system for delivering pulsed ELF-MFs is described in Capone et al. (2009). It consists of a custom-made rectangular, flexible coil kept in place by a Velcro strap and positioned tangential to the inion and to a point 3 cm above nasion. The magnetic pulse generator (B-01; IGEA, Carpi, Italy) supplied the coil with a single-pulsed signal at 75 ± 2 Hz, with a pulse duration of 1.3 ms. The peak intensity of the magnetic field was 1.8 ± 0.2 mT. For sham exposure, the coil was applied in the same position but the pulse generator was not turned on. Subjects were blinded for stimulation conditions. ELF-MFs exposure does not give any sensation; for this reason, it is impossible for the subject to distinguish between real from sham exposure.

### Statistics

The sample size was set to be 25% larger than similar previous studies (Siebner, 2004).

Cortical plasticity was estimated as difference in cortical excitability before and after iTBS. The following measures were therefore computed for both hemispheres:

ΔMEP=preMEP-pre2MEP;pre1

ΔMEP=postMEP-post2MEP1post

where *pre* represents the values obtained before ELF-MFs exposure (*pre1* before iTBS and *pre2* after iTBS) and *post* represents the values recorded after ELF-MFs exposure (*post1* before iTBS and *post2* after iTBS). The effect of ELF-MFs on cortical plasticity was tested with a repeated measure ANOVA for each hemisphere, with two factors: stimulation (two levels: Real and Sham) and Time (Pre and Post). The significance level was set to *p* < 0.05.

## Results

**FIGURE 2:**
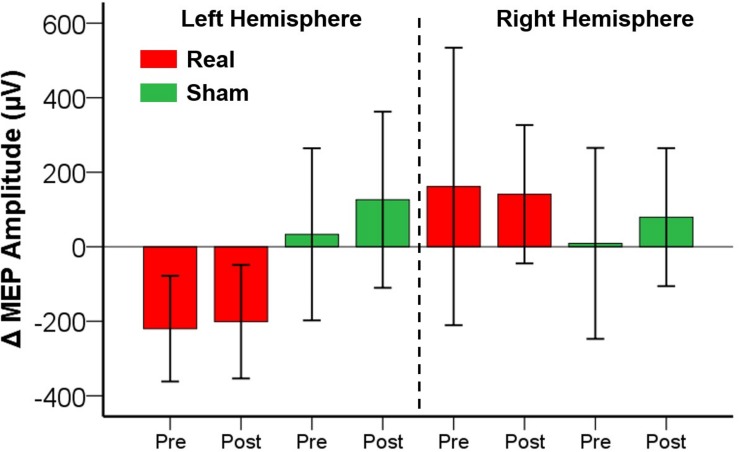
Effects of ELF-MFs on cortical plasticity. ELF-MFs were performed on both hemispheres simultaneously. iTBS was applied over the right hemisphere before (Pre) and after (Post) ELF-MFs. iTBS-related effects were estimated on both right and left hemispheres as MEP amplitude difference post iTBS – pre iTBS (Δ MEP). Each bar represents a Δ MEP. Green bars refer to Sham ELF-MFs, red bars refer to Real ELF-MFs. Inter-subjects’ variability is expressed as 2 standard errors of the mean. No significant effects of ELF-MFs on cortical plasticity were found.

**FIGURE 3:**
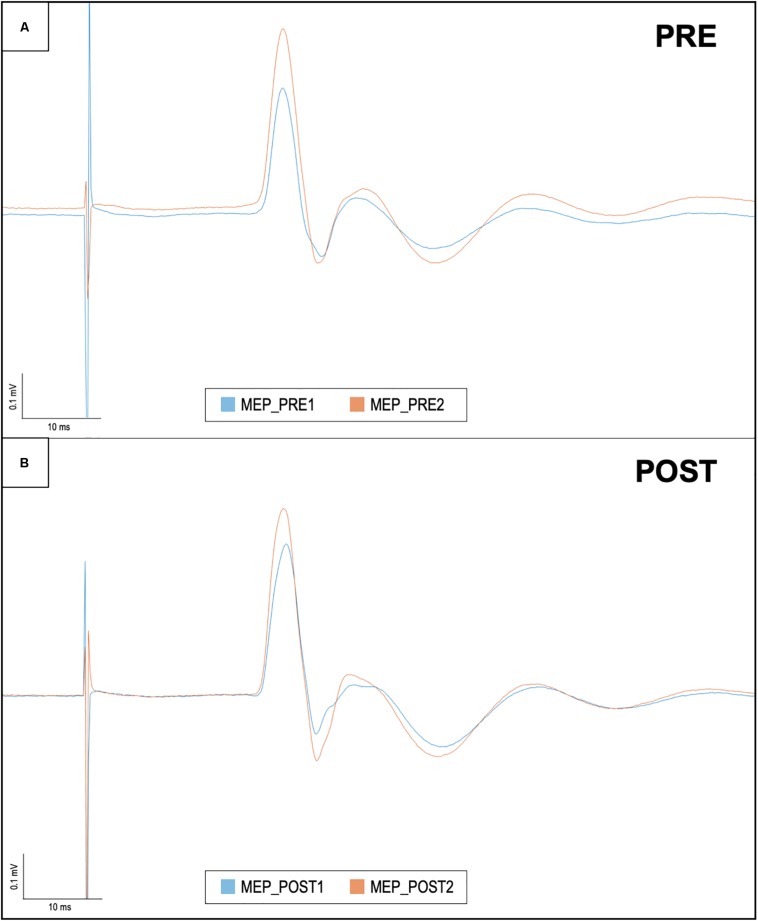
Motor evoked potentials in baseline conditions and after iTBS, before and after ELF-MFs exposure. This figure shows right hemisphere MEP recorded in one representative subject. Each trace is the average of 15 sweeps. **(A)** The baseline evaluation of cortical plasticity: MEP amplitude was measured before (i.e., MEP_PRE1/blue line) and after iTBS (i.e., MEP_PRE2/orange line). After ELF-MFs exposure, the same evaluation was performed again, as shown in **(B)**. Once again, MEP amplitude was measured before (i.e., MEP_POST1/blue line) and after iTBS (i.e., MEP_POST2/orange line).

No adverse events or discomfort sensations were reported. The effects of iTBS and ELF-MFs exposure are illustrated in Figure 2. The database with MEP amplitude raw data is available as Supplementary Material. No significant main factors or interactions were found for both the right and left hemispheres (Figure 3).

## Discussion

This is the first study that has evaluated the effect of ELF-MFs on cortical plasticity in the intact human brain. In a previous study, conducted on a different sample using the same technique, we explored the effect of ELF-MFs on TMS measures of cortical excitability (Capone et al., 2009). We found a pronounced increase in intracortical facilitation (ICF) while other parameters such as RMT, AMT, short-interval intracortical inhibition (SICI) and short-interval afferent inhibition (SAI) remained unchanged. Since ICF mainly reflects excitatory neurotransmission mediated by the NMDA receptors (Di Lazzaro et al., 2008), we hypothesized that ELF-MFs may produce a selective enhancement of glutamatergic activity in human brain.

In the present study we evaluated the effect of ELF-MFs on cortical plasticity, in terms of changes induced by iTBS on TMS measures of cortical excitability. Taken together, our results suggest that although ELF-MFs can influence glutamatergic neurotransmission, they do not affect the propensity of the motor cortex to undergo LTP-like plasticity *in vivo*. Several *in vitro* studies have demonstrated that ELF-MFs modulate the activity of different molecules involved in the mechanisms of LTP such as cAMP (Siebner, 2004), glutamate (Hogan and Wieraszko, 2004), and NMDA receptors (Wieraszko et al., 2005). Moreover, they can influence synaptic plasticity toward an increase (Varró et al., 2009) or a decrease (Balassa et al., 2013) of synaptic strength according to the structural features and neuronal network investigated in the model. Discrepancies between *in vivo* and *in vitro* findings could suggest that the effect of ELF-MFs on synaptic plasticity could be counteracted, *in vivo*, by homeostatic plasticity phaenomena that occur in the intact brain, as already demonstrated for other non-invasive brain stimulation techniques (Müller-Dahlhaus and Ziemann, 2015). This hypothesis is mainly supported by animal studies showing that ELF-MFs exposure has different effects when applied on brain slices as opposed to whole brain (Varró et al., 2009) and by human studies where ELF-MFs modulate glutamatergic neurotransmission without inducing changes in plasticity (Capone et al., 2009).

Other possibilities should be also considered. Since both iTBS and ELF-MFs seem to act on glutamatergic transmission, therefore it is possible that previous exposure to iTBS precludes the development of further plasticity induced by ELF-MFs (ceiling effects/metaplasticity) (Manikonda et al., 2007). Finally, we cannot rule out that the simultaneous excitatory stimulation of both hemisphere by ELF-MFs could produce a null net effect because of an interhemispheric interaction.

Our study has a number of limitations. First, the small sample size of this proof-of-concept study preclude us to draw any definitive conclusions. Second, we explored only the effect of ELF-MFs on motor cortex, while not considering other brain areas (e.g., prefrontal and frontal cortex, somatosensory cortex). Third, the influence of ELF-MFs on cortical plasticity was estimated by measuring the changes in MEP amplitude without considering other important parameters such as input/output curves, RMT, and AMT. Moreover, the number of TMS responses (15) averaged for obtaining MEP amplitude, although in line with previous similar studies (Di Lazzaro et al., 2008, 2011) could be insufficient to capture the effect of ELF-MFs exposure. Finally, we did not perform neuropsychological tests to evaluate the effect of ELF-MFs on cognitive functions related to LTP-like plasticity such as memory or learning processes, but we only relied on neurophysiological measures.

## Conclusion

In conclusion, this pilot study did not unveil any significant effect of 45-min whole-brain exposure to ELF-MFs (75 Hz, 1.8 mT) on LTP-like plasticity in motor cortex. In light of these results, the effects of ELF-MFs on memory and learning reported by some previous studies could depend on: (a) modulation of plasticity mechanisms different from those explored by iTBS (Zhu et al., 2015), (b) selective influence on brain regions specifically involved in memory such as hippocampus (not investigated in this study), (c) the differential characteristics of ELF-MFs used in the different studies. Additional studies in larger sample and with different exposure systems (e.g., different non-invasive brain stimulation techniques protocols) are mandatory to confirm our results and to better understand the effect of ELF-MFs on the human brain.

## Data Availability Statement

The raw data supporting the conclusions of this article will be made available by the authors, without undue reservation, to any qualified researcher.

## Ethics Statement

The studies involving human participants were reviewed and approved by the Campus Bio-Medico Ethical Committee. The patients/participants provided their written informed consent to participate in this study.

## Author Contributions

FC and VD designed the study. FM, MR, and GM acquired the data. GP involved in the analysis of data. FC and FM wrote the manuscript. MR, GM, GP, and VD critically revised the manuscript.

## Conflict of Interest

The authors declare that the research was conducted in the absence of any commercial or financial relationships that could be construed as a potential conflict of interest.

## Supplementary Material

TABLE S1 MEP recordings.
